# *Inter-Ligand* STD NMR: An Efficient 1D NMR Approach to Probe Relative Orientation of Ligands in a Multi-Subsite Protein Binding Pocket

**DOI:** 10.3390/ph15081030

**Published:** 2022-08-21

**Authors:** Serena Monaco, Jonathan Ramírez-Cárdenas, Ana Teresa Carmona, Inmaculada Robina, Jesus Angulo

**Affiliations:** 1 School of Pharmacy, University of East Anglia, Norwich Research Park, Norwich NR4 7TJ, UK; 2 Instituto de Investigaciones Químicas (CSIC—Universidad de Sevilla), 41092 Seville, Spain; 3 Departamento de Química Orgánica, Facultad de Química, Universidad de Sevilla, 41012 Sevilla, Spain

**Keywords:** saturation transfer difference NMR, multi-frequency STD NMR, multi-subsite binding pockets, protein-ligand interactions, ligand-based NMR, Fragment Based Drug Discovery

## Abstract

In recent years, Saturation Transfer Difference NMR (STD NMR) has been proven to be a powerful and versatile ligand-based NMR technique to elucidate crucial aspects in the investigation of protein-ligand complexes. Novel STD NMR approaches relying on “multi-frequency” irradiation have enabled us to even elucidate specific ligand-amino acid interactions and explore the binding of fragments in previously unknown binding subsites. Exploring multi-subsite protein binding pockets is especially important in Fragment Based Drug Discovery (FBDD) to design leads of increased specificity and efficacy. We hereby propose a novel multi-frequency STD NMR approach based on direct irradiation of one of the ligands in a multi-ligand binding process, to probe the vicinity and explore the relative orientation of fragments in adjacent binding sub-sites, which we called *Inter-Ligand* STD NMR (IL-STD NMR). We proved its applicability on (i) a standard protein-ligand system commonly used for ligand-observed NMR benchmarking: Naproxen as bound to Bovine Serum Albumin, and (ii) the biologically relevant system of Cholera Toxin Subunit B and two inhibitors adjacently bound within the GM1 binding site. Relative to Inter-Ligand NOE (ILOE), the current state-of-the-art methodology to probe relative orientations of adjacent ligands, IL-STD NMR requires about one tenth of the experimental time and protein consumption, making it a competitive methodology with the potential to be applied in the pharmaceutical industries.

## 1. Introduction

In the last two decades, ligand-based NMR techniques have been proven to be potent tools to study protein-ligand interactions in drug design [1]. Among them, saturation transfer difference NMR (STD NMR) stands out as one of the most versatile, as it allows not only to efficiently screen fragments libraries, but also to gain structural information on the ligands binding modes, as well as on protein-ligand affinities [2,3,4]. In recent years, novel developments have emerged that expand the standard STD NMR approaches to access further layers of structural information, beyond the classical binding epitope mapping of the ligand, exploiting the inherent inhomogeneity in the process of spin diffusion during the saturation of the protein proton signals [5,6]. This has given rise to the novel concept of multi-frequency STD NMR (i.e., the use of multiple on-resonance frequencies to achieve different outcomes regarding protein saturation), which has proven to be a very efficient way to identify which ligands protons are close to those directly irradiated sources of saturation in the protein binding pocket (e.g., different types of a.a. side chains, irradiated at different on-resonance frequencies in the STD NMR experiments) [7]. Like for standard STD NMR, the concept of multi-frequency STD NMR can be employed to different types of receptor-ligand systems beyond typical protein-ligand interactions, as it has been shown for the interactions of antibiotics with biofilm forming exopolysaccharides [8].

As a paradigmatic example of a multi-frequency STD NMR approach, *DiffErential EPitope mapping* STD NMR (DEEP-STD NMR) [5] demonstrated its ability to provide information about the nature of the amino-acids lining the binding site that are surrounding the ligand in the bound state, whether or not the location of the binding site in the protein surface is already known. If the geometry of the binding pocket is known, the orientation of the ligand within the binding sub-site can be readily inferred. Examples of the strong potential of this novel approach in drug discovery are the application of a DEEP-STD NMR approach to generate an NMR-validated 3D molecular model of a WWP2-inhibitor complex, for the first time [6], and the implementation of a DEEP-STD NMR fingerprinting approach, which allowed to efficiently explore the binding subsites of Cholera Toxin B (CTB), gaining information on the orientation of some novel promising CTB inhibitors in the receptor binding pocket, as well as identifying a hitherto unknown cryptic binding subsite adjacent to the binding pocket of its natural ligand (GM1) [7].

Here, we expand the concept of multi-frequency STD NMR further, by developing a novel tool, *Inter-Ligand* STD NMR (IL-STD NMR), to investigate relative orientation of ligands or molecular fragments bound to multi-subsite binding pockets in protein-ligand ternary complexes. This is particularly relevant for pharmaceutical industry settings implementing Fragment Based Drug Discovery (FBDD) approaches. The initial stages of FBDD focus on binding detection of weak-affinity fragments in adjacent binding subsites of the therapeutic target; their binding affinities and relative orientations are then investigated to build larger, high-affinity binders that would simultaneously occupy all sub-sites. IL-STD NMR relies on a concept similar to selective 1D inter-molecular NOE, yet in the context of a large molecular assembly, and hence with a significant contribution from spin diffusion, comparably to DEEP-STD NMR. For instance, in DEEP-STD NMR, irradiation of different protein protons sitting on different locations within the architecture of the binding pocket, makes ligand protons close to directly irradiated protein protons to show a relative increase in saturation transfer compared to other irradiation frequencies where those protein protons are not directly irradiated [5].

**Figure 1:**
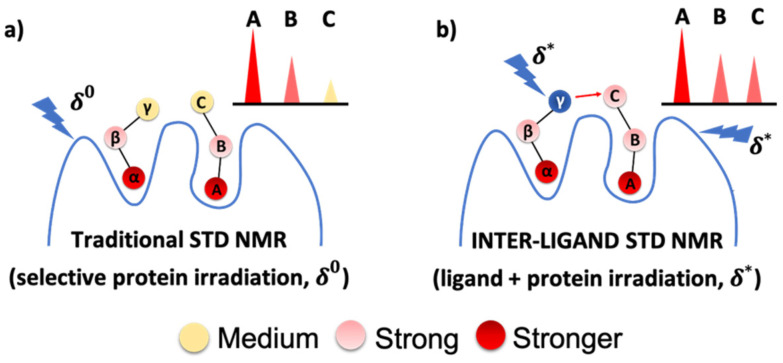
Cartoon representing the IL-STD NMR approach. (**a**) STD NMR with selective irradiation ($\delta^{0}$) on protein protons. (**b**) STD NMR with selective irradiation ($\delta^{*}$ ) on “*reporter ligand*” proton γ (supposed to be close to proton C of the adjacent “*ligand of interest*”) as well as on protein protons. The analysis of the IL-STD NMR experiment is focused exclusively on the protons of the ligand of interest (**A**, **B**, **C** in the cartoon). Significant differences in binding epitope mapping on the ligand of interest will indicate proximity between both ligands.

The aim of IL-STD NMR proposed here is to confirm in a quick way the spatial contacts between adjacent ligands binding to a multi-subsite binding pocket of a given protein based on multi-frequency STD NMR experiments. It does so by elucidating if there are significant changes in the binding epitope mapping of a *ligand of interest*, comparing its STD NMR outcomes from the use of two different irradiation frequencies: (i) a frequency $\delta^{0}$ to achieve a selective protein saturation (standard STD NMR approach), and (ii) a second frequency $\delta^{*}$ to achieve a selective saturation on an adjacent *reporter ligand* (simultaneously saturating the protein), as schematically represented in Figure 1.

This idea originated while investigating the effect of direct irradiation on the determination of a ligand binding epitope mapping from STD NMR, using the Naproxen/Bovine serum albumin (NPX/BSA) complex as a benchmark system. During that study we observed changes in the binding epitope mapping of NPX upon direct ligand irradiation that were not compatible with a simple intra-ligand spin diffusion process in the bound state.

We here aimed to study thoroughly the causes behind those changes, and demonstrate that *inter-ligand saturation transfer* across adjacent binding sites can be observed. We have next applied the proposed IL-STD NMR method to a much more complex and biologically relevant system: the binding of cholera toxin subunit B (CTB) to two adjacent ligands. We prove that IL-STD NMR allows to obtain similar information that can be obtained by Inter-Ligand NOEs (ILOEs) [8] yet in a small fraction (~10%) of the experimental time and protein consumption.

## 2. Results

### 2.1. Observation of IL-STD NMR in a Binary Protein-Ligand Interaction: Naproxen Binding to Bovine Serum Albumin

Noticeably, our first evidence of IL-STD between adjacent subsites in a protein, rather than coming from a protein binding to two ligands, came from a single-ligand one-protein interaction: the binding of Naproxen (NPX) to bovine serum albumin (BSA). The physiological importance of serum albumins and their remarkable ability to bind endogenous and exogenous compounds have determined the usefulness of BSA as a model for the study of protein-ligand interactions [9]. In fact, the complex of BSA with NPX, a non-steroidal anti-inflammatory (Figure S1a), seems to be a very good benchmark system as it has been previously used for optimization of many experimental techniques, particularly ligand-observed NMR experiments [10,11].

We first were using the BSA/NPX system to carry out a simple STD NMR investigation into the impact of direct irradiation of a ligand signal (simultaneously with protein irradiation) on the final binding epitope mapping. To that aim, we ran two STD NMR experiments on the BSA/NPX complex, one irradiating at $\delta^{0}$ = 0.60 ppm (standard selective protein saturation), and the other with $\delta^{*}$ = 1.46 ppm, i.e., irradiating right at the signal of the CH_3_ group at position α to the carbonyl group. The results demonstrate that direct irradiation of a ligand signal in an STD NMR experiment can have a significant impact on the determined binding epitope mapping, with ligand protons spatially close to the irradiated proton showing up to close to 80% increase in relative STD factors (i.e., in the binding epitope; Figure S2). To make a quantitative comparison of the binding epitope mappings at the two frequencies ($\delta^{0}$, $\delta^{*}$), we define the “multi-frequency STD NMR factor” as:

$$\eta {\left(\mathrm{mf}-\mathrm{STD}\right)}_{0}^{*}=\frac{{\mathrm{STD}}^{*}-{\mathrm{STD}}^{0}}{{\mathrm{STD}}^{0}}$$

where “∗” means “direct ligand irradiation” and “0” means standard conditions (selective protein saturation). $\eta {\left(\mathrm{mf}-\mathrm{STD}\right)}_{0}^{*}$ measures the relative changes in binding epitope between the two different irradiation frequencies, i.e., what changes occur in the ligand binding epitope when one of its protons is directly saturated simultaneously with the protein.

**Figure 2:**
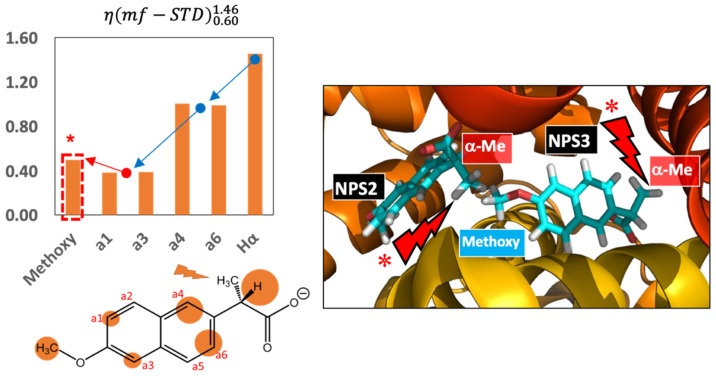
(**Top left**): $\eta {\left(\mathrm{mf}-\mathrm{STD}\right)}_{0.60}^{1.46}$ histogram for the system BSA/NPX at 500 MHz, determined using the initial slopes of the build-up curves acquired at $\delta^{0}$ = 0.60 ppm (standard STD NMR), and $\delta^{*}$ = 1.46 ppm (*on-ligand* methyl group irradiation). The arrows highlight the trend along the NPX molecule (blue indicate decreases in $\eta {\left(\mathrm{mf}-\mathrm{STD}\right)}_{0.60}^{1.46}$, and red increases). The $\eta {\left(\mathrm{mf}-\mathrm{STD}\right)}_{0.60}^{1.46}$ bar for the methoxy protons has been boxed in a dotted square with an asterisk, to highlight the unexpected increase in $\eta {\left(\mathrm{mf}-\mathrm{STD}\right)}_{0.60}^{1.46}$ for the methoxy group, as this is the furthest group from the irradiated moiety. The results report on the proximity of the two bound NPX molecules in the binding sites NPS2 and NPS3, as observed in the crystal structure of the complex (PDB ID: 4OR0, Figure S1a) [12]. In the absence of inter-ligand saturation transfer from NSP2 and NSP3, the $\eta {\left(\mathrm{mf}-\mathrm{STD}\right)}_{0.60}^{1.46}$ values would monotonically decrease from the irradiated methyl group to the furthest methoxy group. (**Bottom left**): $\eta {\left(\mathrm{mf}-\mathrm{STD}\right)}_{0.60}^{1.46}$ values represented on the NPX chemical structure circles of sizes proportional to the $\eta {\left(\mathrm{mf}-\mathrm{STD}\right)}_{0.60}^{1.46}$ values. Spectra and raw data are reported in the Supplementary Materials, Figure S4 and Table S1. (**Right**): NPX binding sites NPS2 and NPS3 in the crystal structure of the BSA/NPX complex, PDB ID: 4OR0 [12]. In the cartoon, red rays indicate the position of the α-methyl protons directly irradiated in the STD NMR experiment ($\delta_{\mathrm{ON}}^{*}$ = 1.46 ppm).

The $\eta {\left(\mathrm{mf}-\mathrm{STD}\right)}_{0.60}^{1.46}$ results represented as a bar graph in Figure 2, left, can be considered arising from a combination of inter-molecular protein-ligand NOEs and intra-molecular ligand NOEs. While the first only takes place in the bound state, the latter occurs in both, the bound and the free state of the ligand. However, the fast chemical exchange required for STD NMR observation leads to very efficient accumulation of saturation in the free state, so that the resulting binding epitope is effectively dominated by the contribution from the bound state (Figure S2b). In this way, irradiation of the chosen methyl signal of NPX leads to significant increases in relative STDs for protons spatially close to that methyl group in the molecule, i.e., protons Hα, a4 and a6 (Figure 2, left).

The trend of $\eta {\left(\mathrm{mf}-\mathrm{STD}\right)}_{0.60}^{1.46}$ values along the molecule (arrows in Figure 2, left) are globally indicative of a reduced effect of the direct irradiation of the methyl group as a function of the distance to the methyl group. Unexpectedly, we observed a disruption in that trend in the form of an increase of the $\eta {\left(\mathrm{mf}-\mathrm{STD}\right)}_{0.60}^{1.46}$ value in the protons of the methoxy group which is well remote from the irradiated methyl protons, both from a spatial point of view and from the point of view of chemical shifts (the methoxy group resonates at 3.91 ppm, while we are irradiating at 1.46 ppm). This observation is not justifiable by intra-ligand spin diffusion: indeed, the histogram in Figure 2, left, clearly show that the $\eta {\left(\mathrm{mf}-\mathrm{STD}\right)}_{0.60}^{1.46}$ values decrease from protons a4 and a6 to protons a1 and a3, in accordance to their distances from the directly irradiated methyl group in α. Therefore, the methoxy group, which is the furthest from the directly irradiated proton should show the smallest $\eta {\left(\mathrm{mf}-\mathrm{STD}\right)}_{0.60}^{1.46}$ value. The fact that this is not true can be only explained by inter-ligand spin diffusion from an adjacently bound ligand, as explained below.

This is indeed what it is observed when inspecting the architecture of the binding pockets for NPX in BSA. Previous structural studies have demonstrated that BSA has three NPX binding sites (NPS1, 2, and 3; Figures S1 and S3) [12,13]. Consequently, the observed STD signals can carry structural information on the binding of NPX to three different sites, which would make very difficult to quantitatively interpret the STD NMR binding epitope. However, all the sites are reported to show different kinetics and affinities (see Supplementary Information and [10,14,15]), so that not all the binding sites will contribute similarly to the intensities of the STD NMR signals. What is more, the observed epitope pattern (Figure S1c) strongly supports that the major contribution indeed comes from the NPX occupying drug site 1 (NPS3 in Figure 2, right). This is the only site in which BSA establishes close hydrophobic contacts with NPX all along the ligand molecule (Figure S3), with both methyl groups showing the lowest amount of saturation transfer, in very good agreement with the NMR observations. In NPS3, the ligand is homogeneously buried within the protein, while the binding modes of Naproxen in NPS1 and NPS2 do not agree with the observed binding epitope (Figure S1c).

In summary, although NPX might be binding to all the three binding sites, the STD NMR responses are mostly reporting on its binding to site NPS3, due to its most favourable fast exchange kinetics between the free and bound states. In Figure 2, right, an expansion around sites NPS2 and NPS3 of the crystal structure of the BSA/NPX complex [16] shows that site NPS3 is adjacent to the NPS2 site. The presence of both ligand molecules in both sites makes the methoxy group of the NPS3-bound NPX molecule account for a very short distance to the α-methyl group of the NPS2-bound NPX molecule (4.4 Å between respective carbon atoms, making all their respective protons to fall within strong NOE distance).

Accordingly, in the STD NMR experiment with direct irradiation of the α-methyl protons of NPX ($\delta^{*}$ = 1.46 ppm), in addition to the intramolecular NOEs in the bound state of NPX leading to strong STD increases in protons α, a4 and a6, the protons of the α-methyl group of the NPS2-bound NPX molecule can very efficiently become extra sources of saturation to the methoxy group of NPS3-bound NPX (Figure 2, right). This is in excellent agreement with the observed pattern of perturbed binding epitope mapping (Figure 2 and Figure S2). Accordingly, the observed relative increase in STD of the NPX methoxy proton does not have an intra-molecular origin, but it is the result of an inter-molecular NOE with the methyl protons of the adjacent ligand molecule in NPS2 (i.e., result from an ILOE). To test the reliability of these observations, we carried out the experiments on the BSA/NPX complex at three different magnetic fields: 500 MHz, 600 MHz and 800 MHz (Figure S5). The inter-molecular NOE character of the observation in the context of the proposed occupancy of adjacent subsites (NPS2 and NPS3) was further confirmed by observation of an intense ILOE between the protons of the methyl and methoxy groups of NPX in a 2D NOESY experiment (Figure S6).

In summary, this study of the single-ligand BSA/NPX system constitutes a solid proof-of-concept of the ability of the novel IL-STD NMR approach to detect inter-ligand proximity in the bound state. Importantly, our study on different magnetic fields strongly supports that this effect is independent from B_0_, so, if resolution allows, the method do not need the use of very high magnetic field spectrometers.

### 2.2. IL-STD NMR to Study Multi-Ligand Binding: Definitions

**Figure 3:**
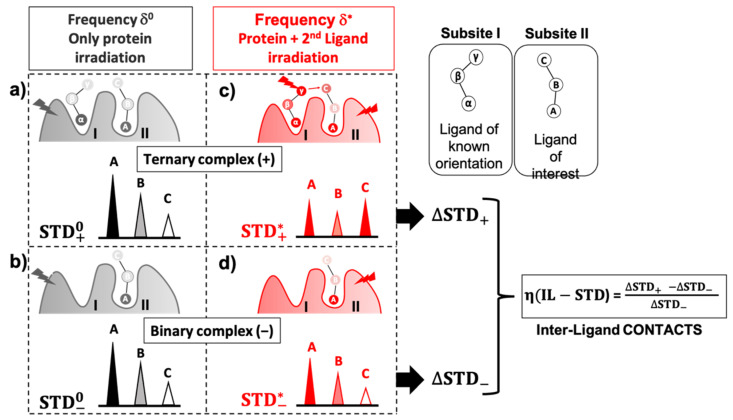
Cartoon representing the IL-STD NMR approach for a system consisting of two ligands binding to a protein in adjacent subsites. (**a**,**b**) In black, STD NMR experiments with selective irradiation (δ^0^) on protein protons, where the experiments are run with the protein containing either both ligands (**a**), ternary complex, “+” giving rise to ${\mathrm{STD}}_{+}^{0}$, or just the *ligand of interest* (**b**), binary complex, “−”, giving rise to ${\mathrm{STD}}_{-}^{0}$. The “+” or “–” sign refers to the presence or absence of the *reporter ligand* of known orientation in the binding subsite-I adjacent to the *ligand of interest* (subsite-II). (**c**,**d**) In red, STD NMR experiments with selective irradiation (δ*) on both, the *reporter ligand* proton γ (expected to be spatially close to proton/s of the *ligand of interest*, e.g., proton C), as well as the protein protons, again with the samples of protein containing either both ligands (**c**), ternary complex, “+”, giving rise to ${\mathrm{STD}}_{+}^{*}$, or just the *ligand of interest* (**d**), binary complex, “−”, giving rise to ${\mathrm{STD}}_{-}^{*}$. Delta-STD values ($\Delta {\mathrm{STD}}_{+}$ = ${\mathrm{STD}}_{+}^{*}$ − ${\mathrm{STD}}_{+}^{0}$ or $\Delta {\mathrm{STD}}_{-}$ = ${\mathrm{STD}}_{-}^{*}$ − ${\mathrm{STD}}_{-}^{0}$ ) identify differences in STD intensities of the *ligand of interest* as a consequence of irradiating at the frequency δ* of the *reporter ligand* in the adjacent subsite, in comparison to δ^0^, whether the *reporter ligand* is present or not, respectively. η(IL−STD) additionally removes potential contributions from the presence or absence of the *reporter ligand* and normalizes values against variations in the binding epitope of the *ligand of interest* simply due to to different levels of protein saturation between δ^0^ and δ*, readily unveiling the existence of inter-ligand contacts in the bound state.

We next decided to explore the applicability of IL-STD NMR to study multi-ligand binding systems, which have high relevance for fragment-based drug discovery approaches. Based on the previous results, our hypothesis was that, in a ternary complex of a protein with two weak-affinity ligands binding to adjacent subsites (one so-called “*reporter ligand*” in, let’s call it subsite-I, and the other the “*ligand of interest*” in subsite-II), a particular multi-frequency STD NMR protocol can be followed to reveal inter-ligand contacts in the bound state, as sketched in Figure 3.

The protocol involves running a total of 4xSTD NMR experiments (two pairs), each one at a distinct saturation frequency: one pair of experiments are run with selective protein irradiation ($\delta^{0}$; black cartoons in Figure 3a,b), and the other pair with selective irradiation ($\delta^{*}$) on some frequency corresponding to a specific proton on the *reporter ligand* (expected to be close to protons of the *ligand of interest*). In the $\delta^{*}$ experiment, saturation is simultaneously generated on protein protons, due to the typically large chemical shifts range and broad signals of a protein (red cartoons, Figure 3c,d). At each frequency, STD NMR experiments are carried out on 2 different samples: a sample containing only the protein and our *ligand of interest* in subsite-II (Figure 3b,d; “binary complex” or “−“ system), and another sample additionally containing the *reporter ligand* whose orientation in the binding pocket is known (in subsite-I; Figure 3a,c; “ternary complex” or “+” system).

For the analysis of IL-STD NMR experiments, the 4 resulting STD NMR outcomes are termed as: ${\mathrm{STD}}^{*}$, resulting from any of the 2 experiments with on-resonance irradiation at the frequency of the *reporter ligand* protons, and ${\mathrm{STD}}^{0}$, resulting from any of the 2 experiments with on-resonance irradiation solely on the protein protons. Additionally, both ${\;\mathrm{STD}}^{*}$ and ${\;\mathrm{STD}}^{0}$ will include subscripts to distinguish the experiments carried out either on the binary (−) or the ternary (+) complex. It is important to highlight that under δ* irradiation most of the protons of the *reporter ligand* will show significant changes in saturation as a consequence of the intra-ligand spin diffusion process in the bound state, as well as the intra-ligand NOE in the free state and, as a consequence, STD intensities on protons of the irradiated ligand will be of no value. In IL-STD NMR experiments, relevant structural information will only be reported for the *ligand of interest* in the ternary complex, by the values of what we now call it the Inter-ligand STD NMR factor, η(IL-STD), obtained as:

$$\eta \left(\mathrm{IL}-\mathrm{STD}\right)=\frac{\Delta {\mathrm{STD}}_{+}^{}-\Delta {\mathrm{STD}}_{-}^{}}{\Delta {\mathrm{STD}}_{-}}$$

where $\Delta {\mathrm{STD}}_{+}$ and $\Delta {\mathrm{STD}}_{-}$ are defined as:

$$\Delta {\mathrm{STD}}_{+}\;=\;{\mathrm{STD}}_{+}^{*}\;-\;{\mathrm{STD}}_{+}^{0}$$

$$\Delta {\mathrm{STD}}_{-}\;=\;{\mathrm{STD}}_{-}^{*}\;-\;{\mathrm{STD}}_{-}^{0}$$

For the sake of simplicity, a more detailed description of this equation is not included here, but in the Supplementary Materials (see also Tables S2 and S3). Large positive η(IL-STD) values on the *ligand of interest* will indicate protons receiving inter-ligand saturation from the irradiated protons of the *reporter ligand* (excluding protons receiving intra-ligand NOE or direct irradiation), thus reporting on proximity between two regions of two bound ligands and confirming that they are occupying adjacent subsites.

**Figure 4:**
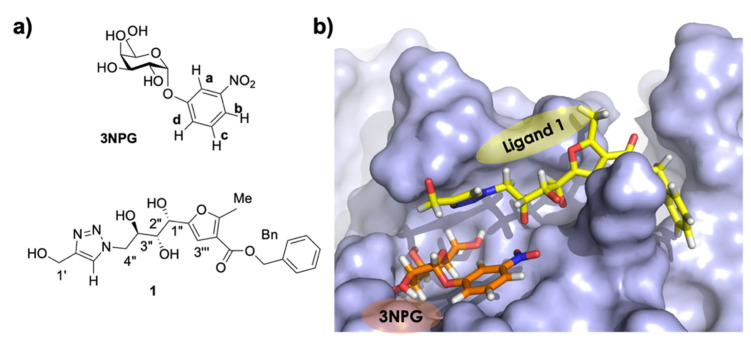
(**a**) Chemical structure and nomenclature of the CTB ligands 3NPG and **1**. (**b**) 3D model of the 3NPG/CTB/**1** ternary complex.

As mentioned above, similar information is available from tr-NOESY experiments on ternary complexes, through the “ILOEs” (Inter-Ligand NOEs) methodology developed by Pellecchia et al. [8]. However, the main advantage of IL-STD NMR compared to ILOE is time efficiency, a major concern for pharmaceutical companies. The long experimental times of tr-NOESY experiments and the complications arising from the acquisition of 2D spectra indeed has made ILOE the least popular among ligand based NMR methods in pharmaceutical companies in a recent poll [16]. Thus, in order to test IL-STD NMR on a biologically and pharmaceutically relevant system, for the next part of this study we chose to prove it on the ternary complex of cholera toxin subunit B (CTB) with two adjacent lead fragments (Figure 4), a protein-ligand system that has been approached by FBDD [17].

### 2.3. IL-STD NMR Applied to Fragments Inhibitors of Cholera Toxin Subunit B (CTB)

We recently carried out a structural study on the binding of several fragments to different subsites of the GM1 binding pocket of CTB [7]. By a combination of DEEP-STD NMR (DEEP-STD fingerprinting), STD NMR competition experiments and computational tools, we unveiled a hitherto unknown binding site adjacent to the two well-known GM1 binding subsites (i.e., the galactose and sialic acid subsites). We demonstrated that CTB is able to form a ternary complex with 3-nitrophenyl-α-D-galactopyranoside (3NPG) and a new inhibitor, **1** (Figure 4a) [17], as proven by the observation of an ILOE cross peak correlating proton Hc,d of 3NPG with proton Htriaz of **1** (Figure S7) [7]. A 3D molecular model of the ternary complex (Figure 4b) was then obtained by superposition of the XRD structure of the CTB/3NPG complex (PDB ID: 1EEI) [18], with a 3D structure of CTB/**1**, obtained by MD simulations validated by CORCEMA-ST [19].

**Figure 5:**
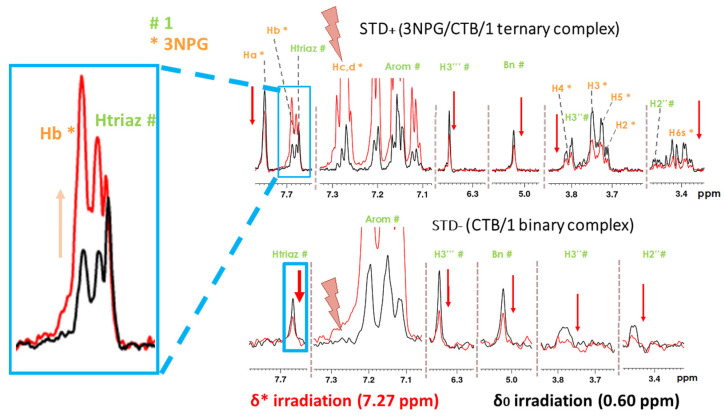
IL-STD difference spectra of (**Top**): the ternary complex 3NPG/CTB/**1** (${\mathrm{STD}}_{+}^{*}$ and ${\mathrm{STD}}_{+}^{0}$ ) and (**Bottom**): the control binary complex CTB/**1** in the absence of 3NPG (${\mathrm{STD}}_{-}^{*}$ and ${\mathrm{STD}}_{-}^{0}$ ), with irradiation at δ^0^ = 0.6 ppm (in black) and at δ* = 7.27 ppm, on resonance at the frequency of protons Hc and Hd of 3NPG (in red). The assignment of all peaks is given and the signal of Htriaz is squared in turquoise and magnified on the left, showing the increased intensity when irradiating at δ* = 7.27 ppm, whereas all the other protons (with the exceptions discussed in the main text) decreased their intensities. A saturation time of 2 s was employed, and a line broadening factor of 0.3 Hz was applied to the FID before FT.

We tested our proposed IL-STD NMR experiments on the 3NPG/CTB/**1** ternary complex. In this case, the orientation of 3NPG in the galactose subsite is known, and hence 3NPG was considered the *reporter ligand*. To probe proximity between protons Hc,d of 3NPG and Htriaz of **1** by IL-STD NMR we then carried out the STD* experiments with irradiation of 3NPG at δ* = 7.27 ppm (Hc,d signal) and the STD^0^ experiments at δ^0^ = 0 ppm, to check if IL-STD NMR could reproduce the information previously obtained by ILOE [7] (Figure 5).

The simple visual inspection of the STD^0^ and STD* spectra in Figure 5 is already very informative. First, in the experiment with irradiation at the aromatic region (δ* = 7.27 ppm), a general decrease of the STD signals was evident, due to the lower number of irradiated protein protons, in comparison with the experiment with aliphatics irradiation. An expected increase was observed for the STD intensities of the Hc,d peak at 7.27 ppm (directly irradiated) and of the aromatic signals of **1** within 0.15 ppm high-field from it (7.21 ppm, 7.16 ppm, 7.12 ppm). Also, the intensity of the Hb proton (7.69 ppm) increased when irradiating the adjacent Hc,d, due to intra-ligand NOE. Notably, proton Htriaz (7.67 ppm) of the ligand of interest, **1**, was the only signal from **1** with an increase in STD intensity, not justifiable by direct irradiation or intra-ligand NOE (blue square, Figure 5). This inter-ligand transfer of magnetization between Hc,d and Htriazole results from their proximity (within NOE distance) in the bound state. The control experiment in the absence of 3NPG (binary CTB/**1** sample), at the bottom section of Figure 5, clearly shows that, in the absence of 3NPG, the signal intensity of Htriaz decreases when irradiating at 7.27 ppm. This is a proof that the increase in STD intensity observed for this proton in the ternary complex, upon irradiation of the reporter ligand, 3NPG, does not arise from any artefact (e.g., neither from direct irradiation of ligand **1**, nor direct irradiation of some protein protons adjacent to **1**), but it is a genuine effect of inter-ligand saturation transfer taking place in the bound state.

**Figure 6:**
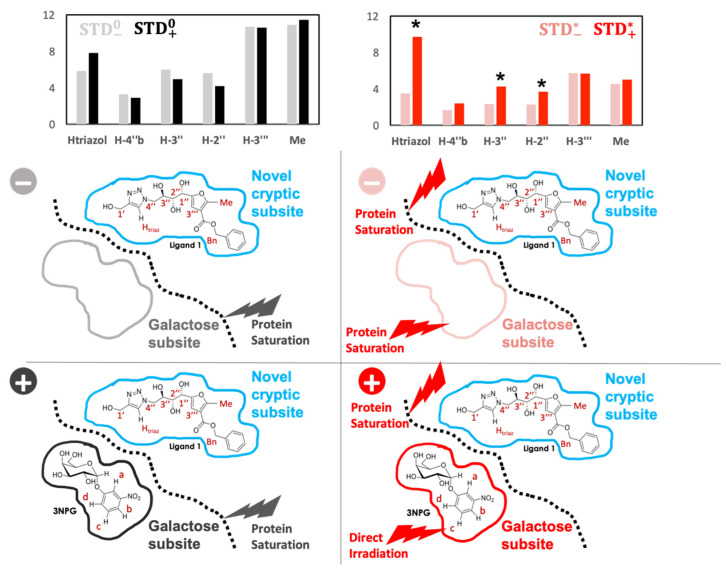
Quantitation of the STD^0^ and STD* results (left and right panels, respectively; δ^0^ = 0 ppm (in black) and at δ* = 7.27 ppm (in red)) for the IL-STD NMR study of the binding of 3NPG and **1** to CTB. Dark and light black bars show the STD^0^ values in samples containing the binary (${\mathrm{STD}}_{-}^{0}\;$, 3NPG absent) or the ternary complex (${\mathrm{STD}}_{+}^{0}$, both ligands present), whereas dark and light red bars show the STD* values in samples containing the binary (${\mathrm{STD}}_{-}^{*}$) or the ternary complex (${\mathrm{STD}}_{+}^{*}$). Asterisks mark the STD intensities significantly increased under direct irradiation of 3NPG. Panels at the bottom represent schematically the 4xSTD NMR experiments. η(IL−STD) values are reported in Table S4. *: direct irradiation.

STD NMR outcomes from the 4xSTD NMR experiments on the CTB system are shown in the top of Figure 6, along with a schematic representation of the experimental configuration of the 4 different experiments sketched on the bottom. Phenyl protons of **1** were excluded from the analysis as direct irradiation could not be fully discarded. Interestingly, just the presence of 3NPG seems to slightly affect the binding epitope of **1**, even when irradiation is selective on the protein (STD^0^ values). However, the variations are small, although, noticeably, only the Htriaz of **1** seemed to increase its STD intensity just as a mere consequence of 3NPG being present in the adjacent subsite. We interpret this result as reinforcing the existence of an ILOE that is able to transfer part of the saturation at δ* irradiation from Hc of 3NPG to Htriaz of **1**.

**Figure 7:**
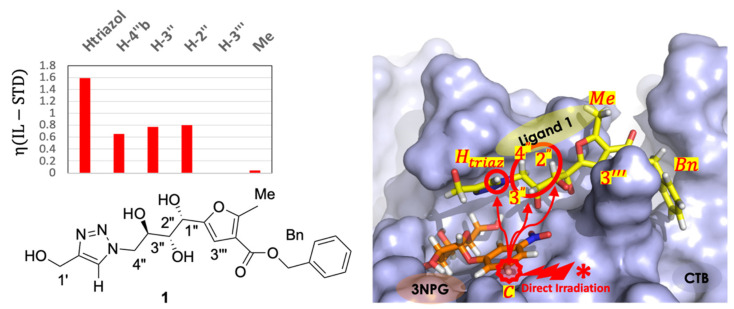
(**Left**): η(IL−STD) factors of protons of **1**, indicating spatial proximity of protons Htriaz, 3″, 2″ and 4″b with proton Hc of 3NPG bound in the adjacent galactose subsite. (**Right**): 3D molecular model of the 3NPG/CTB/**1** ternary complex highlighting the H-H spatial correlations observed by IL-STD NMR.

The detection and quantitation of inter-ligand STDs was carried out through the determination of the η(IL−STD) factor, as defined previously (Figure 3). Monitoring the η(IL−STD) factors of the different protons of **1** clearly showed inter-ligand contacts of protons Htriaz, 4″b, 3″, and 2″ with proton Hc of the 3NPG molecule in the adjacent galactose subsite (Figure 7, left).

## 3. Discussion

The application of the novel IL-STD NMR approach to the benchmark system BSA/NPX, where it was serendipitously detected as a *single-ligand* saturation transfer between adjacent subsites, as well as to the biologically relevant system of CTB in complex with inhibitors bound to adjacent subsites, has shown that the approach is able to confirm inter-ligand proximity in a multi-subsite binding pocket in a highly efficient manner. This type of information is highly relevant to pharmaceutical companies, particularly those interested in the generation of new molecular entities based on the FBDD methodology, where experimental determination of the relative orientations of the initial fragments within the set of subsites in the binding pocket is fundamental to the progression of the identified hits, by appropriately linking chemically the fragments to produce ligands of higher affinity (leads).

It must be highlighted here that similar relevant structural information can be obtained from ILOE experiments though, at this point, it is relevant to make some important comparisons between the two techniques. First, considering experimental time and protein amounts implied by the two approaches (as summarized in Table 1). The IL-STD NMR data acquired for the CTB complexes in this study comprised 4 standard STD NMR experiments, at two frequencies ($\delta^{0}$ and $\delta^{*}$) for the ternary complex 3NPG/CTB/**1**, and the binary complex CTB/**1**, each 30 min long, for a total duration of 2 h. The samples contained a protein-to-ligand ([P]:[L]) ratio of 1:40, with 5 μM of CTB (corresponding to 25 μM binding site concentration, as CTB is a homo-pentamer with one binding site for each sub-domain) and 1 mM of ligand. With 5 μM protein concentration over the 2 × 500 μL IL-STD NMR samples, that accounts for 0.3 mg of protein (CTB is ca. 60 kDa). On the other hand, the ILOE experiments performed on the ternary and the two binary complexes, plus the experiment with the protein alone, took 22 h each, accounting for a total of 88 h (more than 3 days and a half) experimental time in total. Also, the ILOE approach typically implies a low protein to ligand ratio (e.g., 1:10 [P]:[L]), here achieved with 20 μM of CTB (100 μM CTB binding sites) and 1 mM ligand. With 3 × 500 μL ILOE samples, this accounts for 1.8 mg of protein (the 500 μL sample of pure protein used as a control could be re-used for further experiments so it is not included in the protein expenditure calculation). The significant reduction in experimental time and protein consumption put forward IL-STD NMR as a technique of choice for confirmation of inter-ligand proximity in order to validate structural knowledge from theoretical 3D molecular models of protein-ligand ternary complexes (e.g., from docking calculations or MD simulations), especially for systems showing non-overcrowded spectra.

On the other hand, it should be considered that the greatest advantage of ILOE over IL-STD NMR, on the contrary, is that the former does not need any preliminary structural knowledge on the binding of one of the two fragments. In IL-STD NMR, if no structural hint is available, it will be necessary to irradiate all the isolated protons of the spectra to probe if and where any inter-ligand saturation transfer is available, while tr-NOESY experiments do not rely on selective irradiation at all. Still, if STD NMR responses are of high intensity, the IL-STD NMR approach might well be worth it in terms of experimental time. In any case, in comparison with ILOE experiments, the main restriction of IL-STD NMR (and of multi-frequency STD-NMR in general) is that direct irradiation must be avoided on the *ligand of interest*, something that can be especially problematic for crowded spectra.

With the BSA/NPX system we were also interested in answering the question on whether these IL-STD NMR effects need the use of very high field NMR spectrometer (e.g., 800 MHz in this study) to be observed, or the method is equally feasible at lower magnetic fields. The availability of very high field is not always granted, particularly in pharmaceutical industry settings, where most likely medium magnetic fields (e.g., 400, 500 MHz) are more common. The results of the IL-STD NMR experiments carried out at 3 different instruments (500 MHz, 600 MHz and 800 MHz), show that the IL-STD effects are observable with independence of the B_0_ magnetic field, as results were comparable across different instruments.

Finally, the 3NPG/CTB/**1** ternary complex has shown to be a good example of how IL-STD NMR can be carried out also on systems showing highly crowded spectra. Some discussion on technical issues for crowded spectra are however needed. Ideally, on well resolved NMR spectra, IL-STD could be carried out through selective irradiation of protons on any of the two ligands, as long as the selected irradiated proton is spatially close to other protons in the adjacent ligand. In our case, that would mean that irradiation of any of the protons of ligand **1** that showed spatial proximity in the IL-STD NMR experiment with irradiation of Hc,d signal of 3NPG, should equally lead to observation of IL-STD effect on that Hc,d proton of 3NPG in a *reverse* IL-STD NMR approach (i.e., looking for the inter-ligand saturation from ligand **1** to 3NPG instead). However, for ligand **1**, protons Htriaz, H2″, H3″ and H4″ could not be selectively irradiated to test the reverse IL-STD experiment because their NMR signals are in very close proximity to chemical shifts of 3NPG protons (Figure 5). In this case, irradiation on these protons of ligand **1** would lead to contamination of the IL-STD NMR results, due to undesired simultaneous direct irradiation of both ligands. Nevertheless, a control experiment saturating a proton of ligand **1** relatively far from 3NPG, like proton H3′″ (see Figure 7 right), demonstrated no impact on the relative STD NMR intensities of 3NPG (i.e., it did not affect the binding epitope mapping). This demonstrates that IL-STD NMR effects can only be observed for short inter-ligand distances (see Figure S9). In this way, the 3NPG/CTB/**1** system has shown that information on ligand proximity by IL-STD NMR is feasible even in the presence of such high overlap of signals, just by running the selective ligand irradiation on the fragment that shows well isolated signals in the NMR spectrum.

## 4. Materials and Methods

### 4.1. Materials and Sample Preparation

Compounds 3-*nitro*-phenyl α-D-galactopyranoside (3NPG) and Naproxen sodium > 98.8%, cholera toxin subunit B (CTB), bovine serum albumin (BSA), deuterium oxide (99.9% ^2^H), disodium hydrogen phosphate (Na_2_HPO_4_), potassium dihydrogen phosphate (KH_2_PO_4_), sodium chloride (NaCl) and potassium chloride (KCl) were purchased from Sigma (St. Louis, MO, USA). The BSA/NPX sample was composed of 4 mM NPX and 50 μM protein, for a [P]:[L] ratio of 1:80), and analysed at 303 K. The CTB samples were composed of 1 mM ligands and 25 μM binding site concentration (corresponding to 5 μM CTB concentration as CTB is a hetero-pentamer), for a [P]:[L] ratio of 1:40, and analysed at 278 K. All samples were prepared in 10 mM PBS buffer (with 137 mM NaCl and 2.7 mM KCl) at pH 7.4.

### 4.2. Nuclear Magnetic Resonance

For BSA/NPX, the build-up curves were acquired with irradiation frequency of 0.60 ppm (δ^0^) and 1.48 ppm (δ*), at 0.1 s, 0.3 s, 0.5 s, 1.0 s, 1.5 s, 2.0 s saturation times, with 256 scans. For each proton, they were then fitted mathematically to a mono-exponential equation (y = a*[1 − exp(b·x)]), from which the initial slopes (a·b) were obtained. The binding epitope mapping was obtained by dividing the initial slopes by the one of proton a1, to which an arbitrary value of 100% was assigned.

For the CTB samples, STD NMR experiments were acquired with irradiation frequency of δ^0^ = 0.0 ppm (off-reporter ligand) and δ^*^ = 7.27 ppm (on-reporter ligand), at 2 s saturation time, with 128 scans. For all the STD NMR experiments, an STD pulse sequence that included 2.5 ms and 5 ms trim pulses and a 3 ms spoil gradient and water suppression by excitation sculpting with gradients was used (*stddiffesgp.3*). Saturation was achieved applying a train of 50 ms Gaussian pulses (0.40 mW) on the f2 channel, at 0.60 ppm, 1.48 ppm or 7.27 ppm (on-resonance experiments) and 40 ppm (off-resonance experiments). The broad protein signals were removed using a 40 ms spinlock (T_1ρ_) filter (*stddiff.3*). Due to the strong overlapping observed in the CTB ternary complex, CTB samples were all analyzed at a ^1^H frequency of 800.23 MHz on a Bruker Avance III spectrometer equipped with a 5 mm probe TXI 800 MHz H-C/N-D-05 Z BTO. BSA/NPX experiments were recorded at ^1^H frequency of (i) 499.68 MHz on a Bruker Avance NEO spectrometer equipped with a 5 mm iProbe PA QXI 600S3 H-C/N/F-D-05 Z, (ii) 600 MHz on a Bruker Avance III spectrometer equipped with a cryoprobe QCI Cryo 5mm (1H/19F 15N/13C) for ^1^H, ^15^N, ^13^C, and ^19^F with ^2^H decoupling, (iii) 800.23 MHz on a Bruker Avance III, as used for CTB experiments.

## 5. Conclusions

We report hereby for the first time the proof-of-concept that *Inter-Ligand* saturation transfer NMR (IL-STD NMR) is technically feasible to probe inter-ligand proximity between bound ligands in adjacent binding sites of a given protein. This is an important proof of principle that opens the doors to quick and easy validation of 3D theoretical molecular models of protein-ligand ternary complexes, confirming relative orientations between ligands binding to two adjacent subsites in a given protein binding pocket. While IL-STD NMR is remarkably more time- and cost-efficient than ILOE, which is an important feature for model validation in fragment-based drug discovery, data interpretation requires consideration of potential risks (e.g., impact of direct irradiation must be pondered and mitigated by the acquisition of control spectra). We envision that IL-STD NMR, despite those potential limitations, can become a useful tool to confirm in a fast and efficient fashion structural hypothesis about orientation of two bound fragments, considerably aiding the fragment-based drug discovery process in pharmaceutical settings and proving once more the great potential of multi-frequency STD NMR.

## Figures and Tables

**Table 1:** Comparison between the experimental time and protein investment needed for the IL-STD vs. ILOE approach for the study of relative orientation of fragments bound to CTB.

	Experimental Time	Protein Amount
IL-STD NMR	2 h	0.3 mg
ILOE	88 h (ca. 3.5 days)	1.8 mg

## Data Availability

Data is contained within the article and Supplementary Materials.

## Supplementary Materials

The following supporting information can be downloaded at: https://www.mdpi.com/article/10.3390/ph15081030/s1, Figure S1: BSA distribution of binding sites and NPX build-up curves; Figure S2: Binding epitope mapping of NPX at two different irradiation frequencies; Figure S3: NPX-BSA contacts in different binding sites; Figure S4: STD NMR spectra NPX/BSA; Figure S5: IL-STD factors for NPX at different magnetic fields; Figure S6: NOESY experiment of the NPX/BSA sample; Figure S7: ILOE of the binding of ligand 1 and 3NPG to CTB; Figure S8: IL-STD NMR control experiments on binary complexes; Figure S9: negative IL-STD NMR control in the ternary complex; Table S1: IL-STD raw data BSA/NPX; Table S2: Definitions of STD NMR factors under different irradiation frequencies; Table S3: Definitions of IL-STD NMR factors; Table S4: Raw STD intensities 3NPG/CTB/1 ternary complex.
