# Supplementary material for: Inter-Ligand STD NMR: An Efficient 1D NMR Approach to Probe Relative Orientation of Ligands in a Multi-Subsite Protein Binding Pocket
Source: Pharmaceuticals (Basel). 2022 Aug 21;15(8):1030. doi: 10.3390/ph15081030 (PMC9415034; doi:10.3390/ph15081030)
Supplement: Supplementary file 1 [file pharmaceuticals-15-01030-s001.zip › pharmaceuticals-1776355-Supplementary Material 2.pdf]

Inter-Ligand STD NMR: An Efficient 1D NMR Approach to Probe Relative Orientation of Ligands in a Multi-subsite Protein Binding Pocket

Serena Monaco,[1] Jonathan Ramírez-Cárdenas,[2] Ana T. Carmona,[3] Inmaculada Robina,[3] Jesús Angulo[1,2]*

[1] School of Pharmacy, University of East Anglia, Norwich Research Park, NR4 7TJ Norwich, UK
[2] Instituto de Investigaciones Químicas (CSIC – Universidad de Sevilla), 41092 Seville, Spain
[3] Departamento de Química Orgánica, Facultad de Química, Universidad de Sevilla, 41012-Sevilla, Spain
* Correspondence: j.angulo@iiq.csic.es

Supporting information table of content

- **(S1) Binding epitope mapping of NPX supports main contribution from "drug site 1" in BSA**
- **(S2) Impact on STD NMR derived epitope mapping upon direct irradiation of the ligand: the case of NPX binding to BSA**
- **(S3) IL-STD raw data and spectra of the BSA/Naproxen complex**
- **(S4) Comparison of IL-STD NMR experiments at different B_0 (500 MHz, 600 MHz and 800 MHz) for BSA/NPX**
- **(S5) IL-STD factor ($\eta(IL-STD)$) in multi-ligand systems and data processing protocol.**
- **(S6) ILOEs and IL-STD experiments for the CTB ternary complex**
- **(S7) IL-STD raw data for CTB ternary complex**

(S1) Binding epitope mapping of NPX supports main contribution from "drug site 1" in BSA

Naproxen occupies three well characterised binding sites of BSA: "drug site 2", "drug site 1" and "fatty acid site 6", the latter two being adjacent to each other (Figure S1a) [1],[2]. Defining binding affinities to each of the three sites is controversial and many K_D values for this system have been published, ranging from 0.034 to 0.82 μM [3],[4],[5].

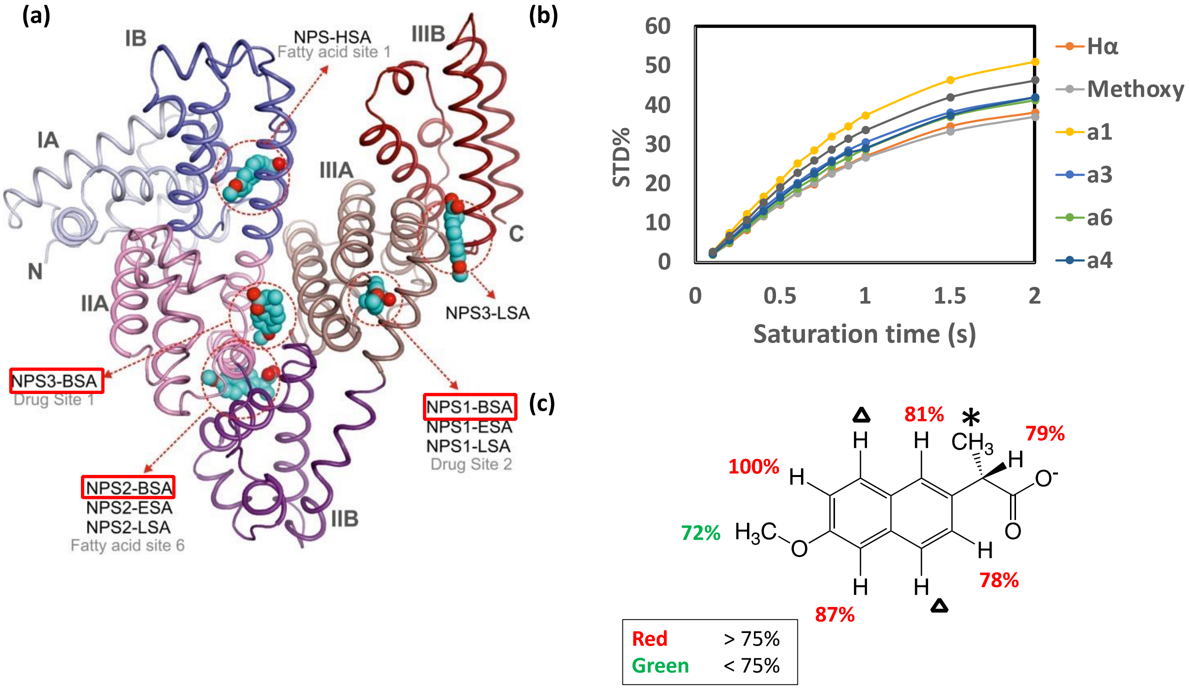

Figure S1. (a) Distribution of Naproxen (NPS) binding sites in bovine (BSA), equine (ESA), leporine (LSA) and human (HSA) serum albumin complexes, from [1]. The three BSA binding sites of relevance for this work are highlighted in red. **(b)** STD NMR build-up curves for Naproxen in complex with BSA (800 MHz). **(c)** STD NMR binding epitope mapping (800 MHz).

STD NMR analysis of the BSA/Naproxen complex shows a binding epitope map that is rather homogeneous (Figure S1c) with the lowest relative STD values on two non-aromatic sets of protons (the methoxy group, and the proton in α to the carboxylic group,) while the strongest value is on proton a1, the aromatic proton in *ortho* to the methoxy group. The observed STD signals result from the combination of Naproxen binding in the three different sites, most likely with different kinetics and affinities. This makes very cumbersome to accurately interpret the STD NMR binding epitope. Nevertheless, the observed epitope pattern (Figure S1c) strongly supports that the major contribution comes from the Naproxen occupying drug site 1 (NPS3 in Figure S1a). Naproxen establishes close contacts with BSA all along the ligand molecule (Figure S1xc), with both methyl groups showing the lowest amount of saturation transfer, agreeing with the binding mode of NPX in NPS3 in the crystal structure (Figure S3), where the ligand is homogeneously buried within the protein, leaving the non-aromatic ends slightly more solvent exposed, while the binding modes of Naproxen in NPS1 and NPS2 look much less compatible with the observed binding epitope (Figure S3).

(S2) Impact on STD NMR derived epitope mapping upon direct irradiation of the ligand: the case of NPX binding to BSA

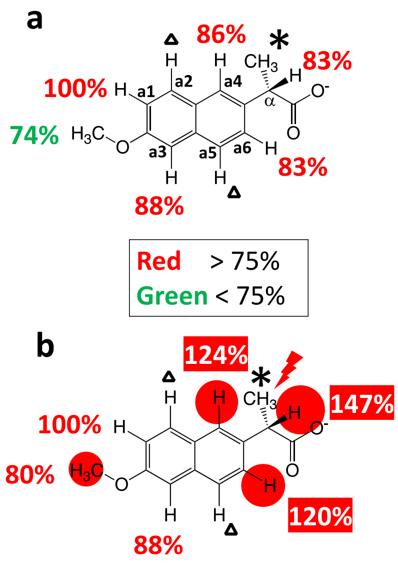

Figure S2. Binding epitope mapping of NPX for its interaction with BSA obtained at two different "on-resonance" irradiation frequencies: **(a)** δ^0= 0.6 ppm, i.e. a standard STD NMR approach with selective protein saturation, and **(b)** δ^*= 1.46 ppm, simultaneously irradiating the protein and methyl protons at α position from the carbonyl group (labelled with asterisk). Both epitopes show STD relative values after normalization against the same STD intensity (a1 proton), to highlight the increase in saturation transfer for NPX protons upon irradiation of the methyl ligand group.

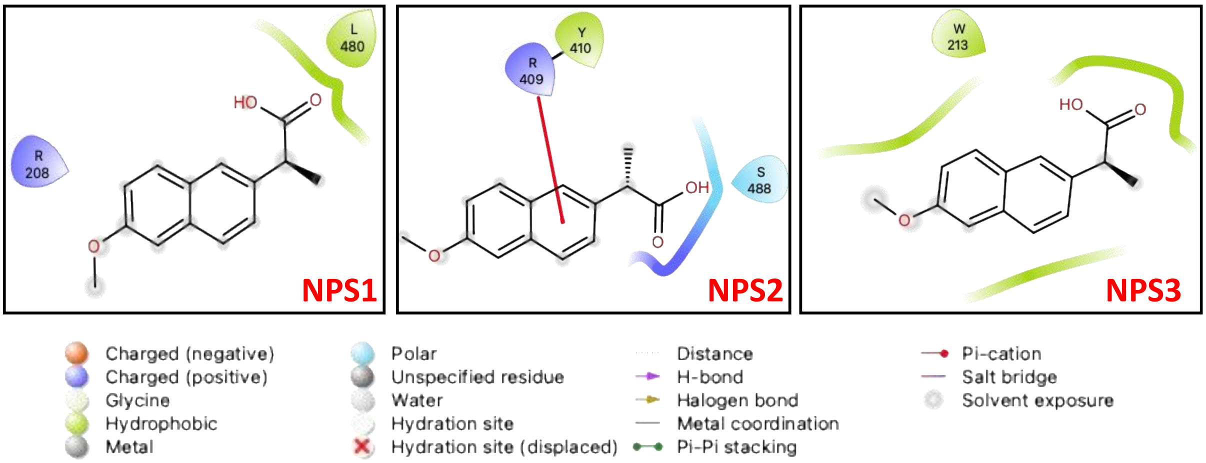

Figure S3. Diagrams of NPX-BSA contacts in the three different NPX binding sites of BSA: NPS1 (left), NPS2 (centre), and NPS3 (right). Comparison with the binding epitope mapping of NPX in Figure S1c supports that observed STD NMR intensities have a main contribution from the binding of NPX to NPS3, where the protein establishes close hydrophobic contacts all along the ligand molecule, with both methyl groups showing the lowest amount of saturation transfer.

(S3) IL-STD raw data and spectra of the BSA/Naproxen complex

Proton	^{1}H δ (ppm)	500 MHz Initial slope δ^0	500 MHz Initial slope δ^*	500 MHz $\eta(\mathrm{mf}-\mathrm{STD})_{0.6}^{1.46}$	600 MHz Initial slope δ^0	600 MHz Initial slope δ^*	600 MHz $\eta(\mathrm{mf}-\mathrm{STD})_{0.6}^{1.46}$	800 MHz Initial slope δ^0	800 MHz Initial slope δ^*	800 MHz $\eta(\mathrm{mf}-\mathrm{STD})_{0.6}^{1.46}$
**Methoxy**	3.91	42.9	64.2	0.50	123.2	139.2	0.13	47.3	62.9	0.33
**a1**	7.18	57.9	80.1	0.38	155.0	168.2	0.09	65.5	85.0	0.30
**a3**	7.33	50.7	70.3	0.39	132.0	144.3	0.09	57.3	67.6	0.18
**a4**	7.73	49.6	99.4	1.00	136.9	177.4	0.30	53.2	103.0	0.94
**a6**	7.44	48.3	96.0	0.99	128.4	163.3	0.27	51.1	99.2	0.94
**Hα**	3.75	48.0	117.8	1.45	138.8	194.4	0.40	51.6	120.3	1.33

Table S1. Values of the multi-frequency STD NMR factor $\eta(\mathrm{mf}-\mathrm{STD})_{0.6}^{1.46}$ for the BSA/NPX complex, at 3 different magnetic fields, determined using the initial slopes of the build-up curves acquired at δ^0 = 0.60 ppm (only protein saturation) and δ^* = 1.46 ppm (protein and ligand saturation).

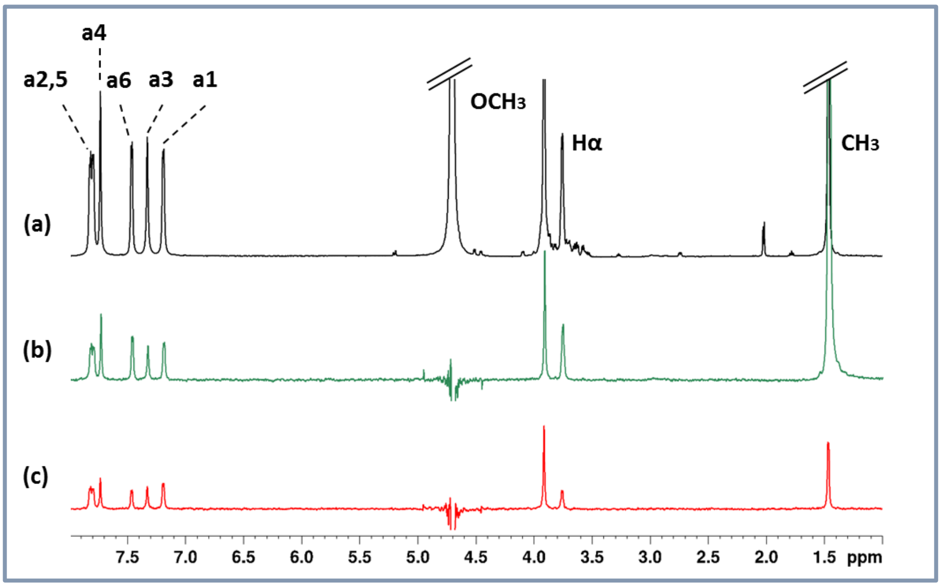

Figure S4. 4 mM of Naproxen in the presence of 50 μM BSA in deuterated PBS buffer, pH 7.4, 303 K, saturation time 1 s. **(a)** Reference spectra (x 1); **(b)** STD NMR spectrum with on-resonance irradiation at 1.46 ppm (x 32); **(c)** STD NMR spectrum with irradiation at 0.60 ppm (x 32).

(S4) Comparison of IL-STD NMR experiments at different B_0 (500 MHz, 600 MHz and 800 MHz) for BSA/Naproxen

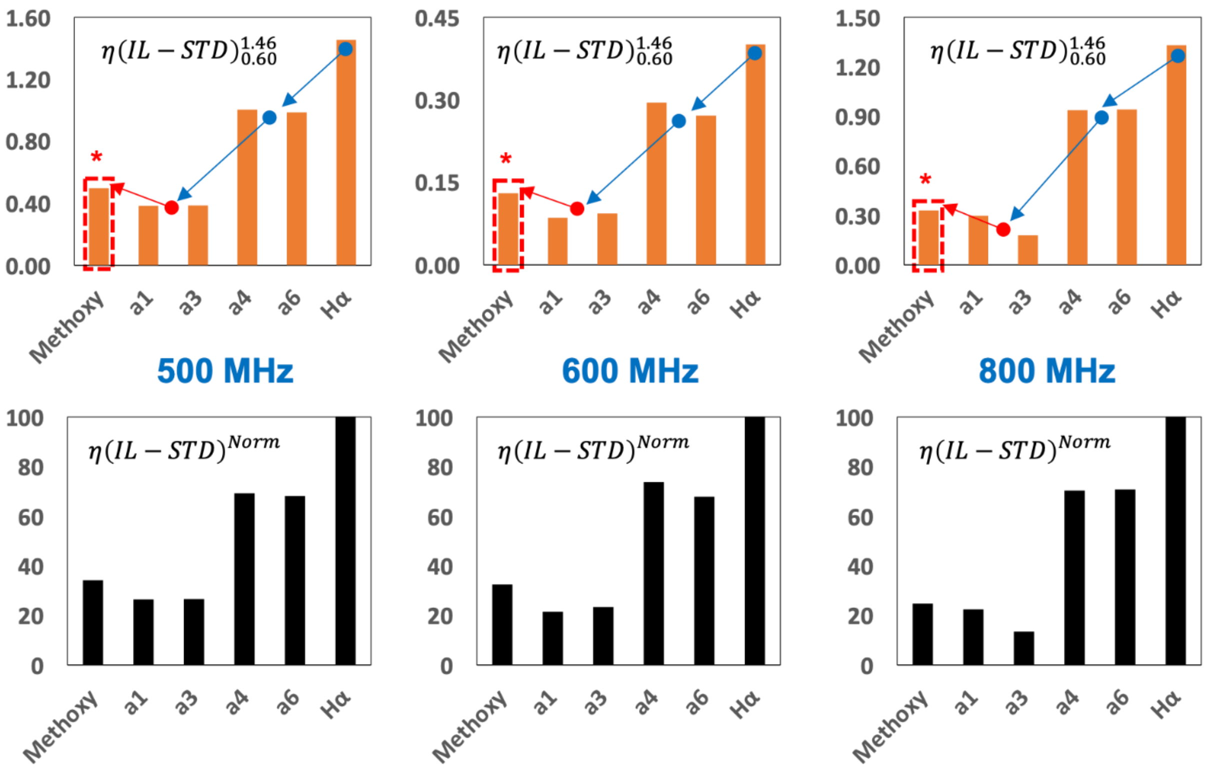

Figure S5. Top: η(IL-STD) histograms for the system BSA/Naproxen at 500, 600, and 800 MHz, determined using the initial slopes of the build-up curves acquired at δ^0 = 0.60 ppm (only protein saturation) and δ^* = 1.46 ppm (protein and ligand saturation). The arrows highlight the trend along the NPX molecule (blue indicate decreases in $\eta(mf-STD)_{0.60}^{1.46}$, and red increases). The $\eta(mf-STD)_{0.60}^{1.46}$ bar for the methoxy protons has been boxed in a dotted square with an asterisk, to highlight the unexpected increase in $\eta(mf-STD)_{0.60}^{1.46}$ for the methoxy group, as this is the furthest group from the irradiated moiety. **Bottom:** η(IL-STD) normalised in a 0-100 % scale, to highlight the similarity in relative terms among different spectrometer magnetic fields.

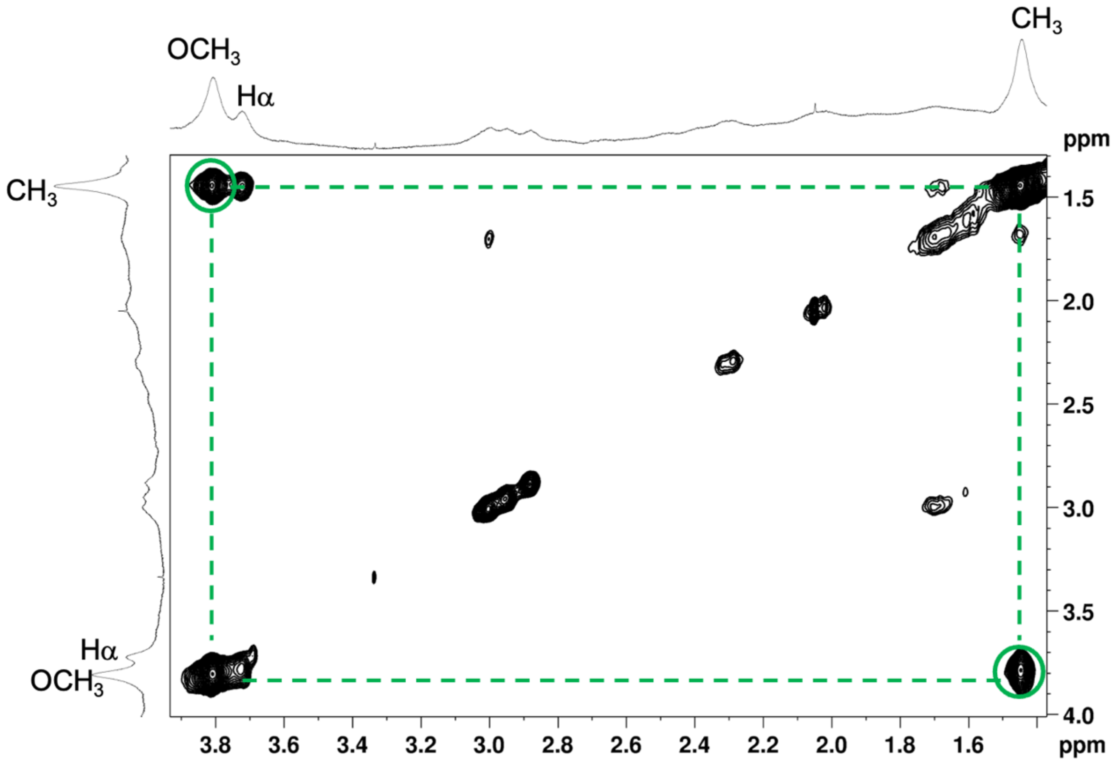

Figure S6. Top: Expansion of the 2D NOESY experiment (mixing time 600 ms, 500 MHz, 303 K) of the sample of BSA (400 uM; 3 x NPX binding sites = 1.2 mM in binding sites) with NPX (12 mM). Highlighted in green the ILOE cross peak between the methyl and the methoxy groups of NPX, allocated in opposite sides of the molecule. The NOESY confirms the IL-STD NMR results supporting the observation of an inter-ligand NOE between adjacent subsites.

(S5) IL-STD factor ($\eta(IL - STD)$) in multi-ligand systems and data processing protocol.

Like other multi-frequency STD NMR approaches (e.g. DEEP-STD NMR), IL-STD NMR relies on acquiring pairs of STD NMR experiments at two irradiation frequencies and quantifying their differences in *relative* STD intensities. In IL-STD NMR, we distinguish between the "*ligand of interest*", aim of the study, and the "*reporter ligand*", which has a known orientation in the binding subsite adjacent to the *ligand of interest*; we call the two different STD outcomes as:

- $\boldsymbol{STD^*}$**,** when we irradiate on resonance protons of the "*reporter ligand*" (frequency δ^*, hitting at the same time the protein too), and
- $\boldsymbol{STD^0}$, when we irradiate on the protein solely (frequency δ^0, standard STD NMR approach).

For ternary complexes in which our "*ligand of interest*" is binding to a subsite (let's call it *subsite-II*) near in space to another one (*subsite-I*) in which the binding orientation of a second different ligand is known ("*reporter ligand*"), the whole IL-STD NMR analysis involve the use of two different samples:

(i) one sample containing only the protein and our *ligand of interest* in *subsite-II* and

(ii) another sample additionally containing the *reporter ligand* (in *subsite-I*) whose orientation in the adjacent binding subsite is known. The STD results from these samples are named as in Table S2

**STD NMR at Frequency δ***		**STD NMR at Frequency δ^0**	
**STD factor**	**Description**	**STD factor**	**Description**
STD_+^*	- (*) Irradiation on-ligand & on-protein<br>- (+) Reporter ligand present (subsite-I)	STD_+^0	- (0) Irradiation on-protein<br>- (+) Reporter ligand present (subsite-I)
STD_-^*	- (*) Irradiation on-ligand & on-protein<br>- (-) Reporter ligand absent	STD_-^0	- (0) Irradiation on-protein<br>- (-) Reporter ligand absent

Table S2. Definitions of STD NMR factors obtained under direct *reporter ligand* irradiation (left columns) and under protein irradiation (right columns). The experiments are done on two samples: one containing only the protein and *ligand of interest* (sample "-", bottom row), and the other containing the protein in the presence of both, the *ligand of interest* and the *reporter ligand* (sample "+", top row).

When comparing the STD intensities of the *ligand of interest* (*subsite-II*) under irradiation of the protein alone, frequency δ^0, against irradiation of both the *reporter ligand* and the protein, frequency δ*, we need to take into account two possible sources of impact in the binding epitope mapping of our ligand, besides the inter-ligand transfer of saturation, which are:

i. Impact of different protein saturation levels between irradiation frequencies δ^0 and δ^*

ii. Impact of the presence of the reporter ligand in the adjacent subsite (*subsite-I*)

To purely identify inter-ligand transfer of saturation, we need to remove these potential contributions. The analysis of the experiments is proposed in Table S3:

**Measurement**	**Description**
$\Delta STD_{+} = STD_{+}^{*} - STD_{+}^{0}$ (*reporter ligand* present)	Reports on:<br>- Impact on the *ligand of interest* due to saturation of *reporter ligand* (IL-STD)<br>- Impact on the *ligand of interest* due to different levels of protein saturation between δ^* and δ^0
$\Delta STD_{-} = STD_{-}^{*} - STD_{-}^{0}$ (*reporter ligand* absent)	Reports on:<br>- Impact on the *ligand of interest* due to different levels of protein saturation between δ^* and δ^0
$\Delta\Delta STD = \Delta STD_{+} - \Delta STD_{-}$	Reports on:<br>- Impact on the *ligand of interest* due to saturation of the *reporter ligand* (IL-STD). Reports on Inter-Ligand contacts in the bound state.
$\eta(IL - STD) = \frac{\Delta\Delta STD}{\Delta STD_{-}}$	**<u>IL-STD FACTOR:</u>** Normalised Double Delta STD with respect to the impact binding epitope mapping due of the *ligand of interest* to different levels of protein saturation between δ^* and δ^0.

Table S3. Definitions of IL-STD NMR factors

(S6) ILOEs and IL-STD experiments for the CTB ternary complex

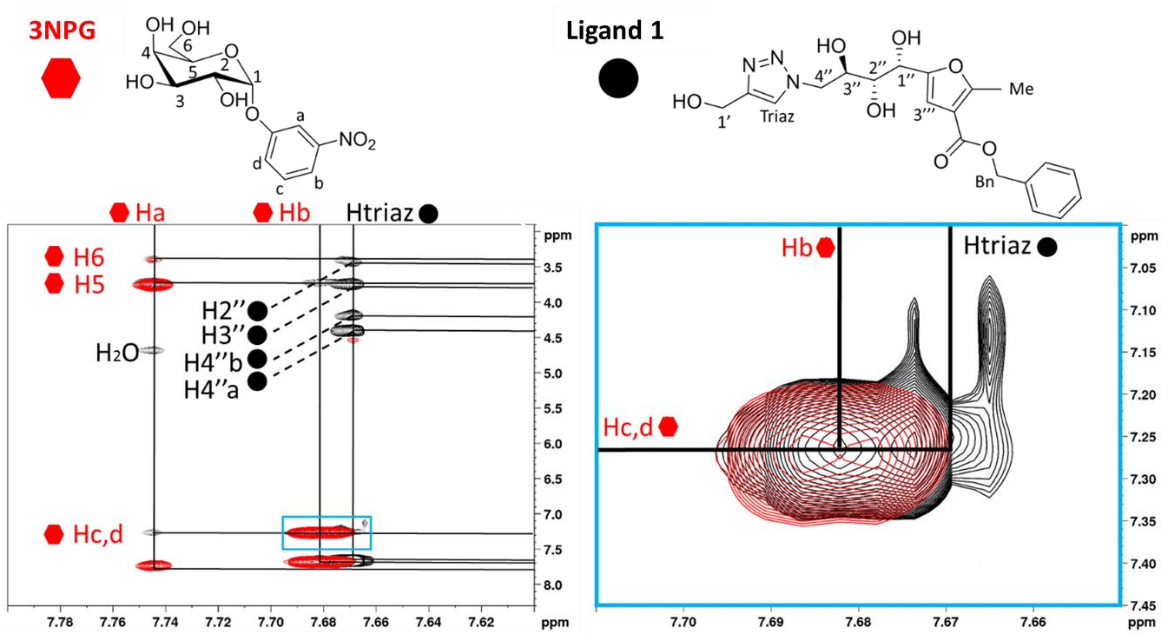

Figure S7. Left, expansion of the tr-NOESY experiments of the ternary complex 3NPG/CTB/**1** (in black) and the control CTB/3NPG (in red). Diagonal and cross peaks are assigned and the ILOE signal between Hc,d of 3NPG and Htriazole of **1** is squared in turquoise and magnified on the right. 3NPG frequencies are highlighted by red hexagons, and ligand **1** frequencies are highlighted by black circles. The structure and nomenclature of the two ligands is reported here again for clarity.

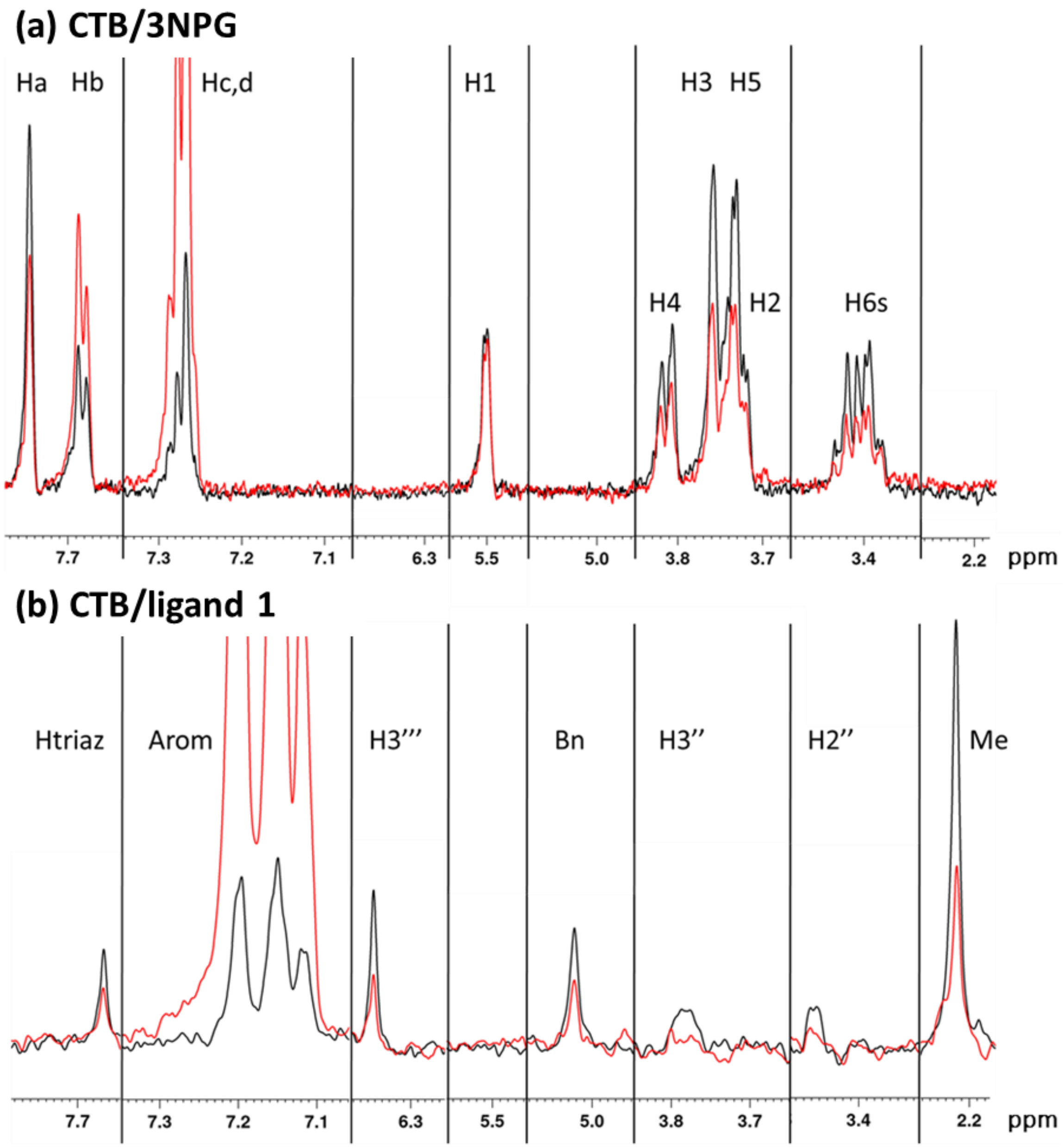

Figure S8. IL-STD control experiments on **(a)** 3NPG and **(b) 1** in a binary complex with CTB. Difference spectra with irradiation frequency of δ^0=0.6 ppm are shown in black, and difference spectra with direct irradiation at δ^*=7.27 are shown in red.

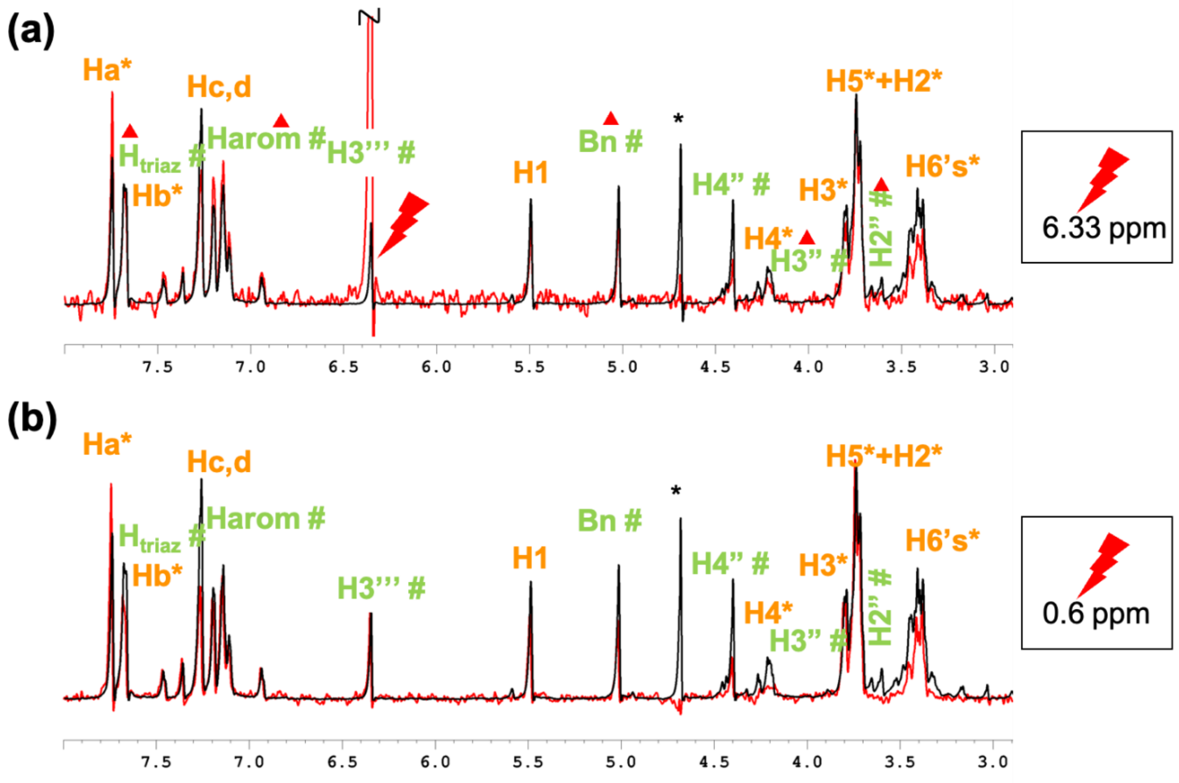

Figure S9. IL-STD NMR control experiments on the sample of the ternary complex 3NPG/CTB/**1**. **(a)** STD NMR (t_{sat} = 2 s) with irradiation frequency at δ^*= 6.36 ppm (H3''' of **1**). **(b)** STD NMR (t_{sat} = 2 s) with irradiation frequency at δ^0 = 0.6 ppm. In (a) the red triangles highlight those protons of ligand **1** that increase STD intensities as a consequence of the direct irradiation of the H3''' in that ligand. Comparison of the intensities of the STD signals of 3NPG between both spectra demonstrate that there is no IL-STD effect upon irradiation of H3''', as that region of ligand **1** is far away to the protons of 3NPG (see Figure 2 in the main text).

(S7) IL-STD raw data for CTB ternary complex

		3NPG/CTB/1 (ΔSTD_+)			CTB/1 (ΔSTD_-)			
^{1}H δ	Atom ID	STD% (δ^0)	STD% (δ*)	ΔSTD_+	STD% (δ^0)	STD% (δ*)	ΔSTD_-	$\eta(IL-STD)$
**2.24**	**Me**	11.46	5.02	-0.56	10.91	4.54	-0.58	0.04
**3.48**	**H-2''**	4.19	3.69	-0.12	5.62	2.28	-0.59	**0.80**
**3.79**	**H-3''**	4.95	4.26	-0.14	5.99	2.33	-0.61	**0.77**
**4.23**	**H-4''b**	2.9	2.41	-0.17	3.28	1.67	-0.49	**0.66**
**5.03**	**CH_2(Bn)**	6.97	3.29	-0.53	6.14	3.45	-0.44	-0.21
**6.36**	**H-3'''**	10.61	5.68	-0.46	10.7	5.73	-0.46	0.00
**7.12**	**H para**	9.16	42	3.59	11.57	52	3.49	-0.03
**7.16**	**H meta**	11.13	54	3.85	10.5	57	4.43	0.13
**7.21**	**H orto**	9.25	65	6.03	10.15	73	6.19	0.03
**7.68**	**Htriazol**	7.83	9.71	0.24	5.84	3.48	-0.40	**1.59**

Table S4. Raw STD NMR intensities for the ternary (3NPG/CTB/**1**) and the binary (CTB/**1**) complexes. The STDs at δ^0=0.6 ppm and STDs at δ^*=7.27 ppm, ΔSTDs as well as the calculated $\eta(IL-STD)$ are shown. Protons of **1** with the largest $\eta(IL-STD)$ values are highlighted in red.

References

1. Bujacz, A., K. Zielinski, and B. Sekula, *Structural studies of bovine, equine, and leporine serum albumin complexes with naproxen.* Proteins: Structure, Function, and Bioinformatics, 2014. **82**(9): p. 2199-2208.
2. Simard, J.R., et al., *Location of high and low affinity fatty acid binding sites on human serum albumin revealed by NMR drug-competition analysis.* Journal of molecular biology, 2006. **361**(2): p. 336-351.
3. Fielding, L., S. Rutherford, and D. Fletcher, *Determination of protein–ligand binding affinity by NMR: observations from serum albumin model systems.* Magnetic resonance in Chemistry, 2005. **43**(6): p. 463-470.
4. Honoré, B. and R. Brodersen, *Albumin binding of anti-inflammatory drugs. Utility of a site-oriented versus a stoichiometric analysis.* Molecular pharmacology, 1984. **25**(1): p. 137-150.
5. Maruthamuthu, M. and S. Kishore, *Binding of naproxen to bovine serum albumin and tryptophan-modified bovine serum albumin.* Journal of Chemical Sciences, 1987. **99**(4): p. 273-279.
